# Lean mass and biological maturation as predictors of muscle power and strength performance in young athletes

**DOI:** 10.1371/journal.pone.0254552

**Published:** 2021-07-12

**Authors:** Paulo Francisco de Almeida-Neto, Rafaela Catherine da Silva Cunha de Medeiros, Dihogo Gama de Matos, Adam D. G. Baxter-Jones, Felipe J. Aidar, Gilmara Gomes de Assis, Paulo Moreira Silva Dantas, Breno Guilherme de Araújo Tinôco Cabral

**Affiliations:** 1 Health Sciences Center—Department of Physical Education, Federal University of Rio Grande do Norte, DEF-UFRN, Natal, RN, Brazil; 2 Health Sciences Center, Federal University of Rio Grande do Norte, CCS-UFRN, Natal, RN, Brazil; 3 Department of Physical Education—State University of Rio Grande do Norte, UERN, Mossoró, RN, Brazil; 4 Cardiovascular & Physiology of Exercise Laboratory, Faculty of Kinesiology and Recreation Management, University of Manitoba, Winnipeg, Canada; 5 College of Kinesiology, University of Saskatchewan, Saskatoon, Canada; 6 Department of Physical Education, Federal University of Sergipe, UFS, São Cristovão, SE, Brazil; 7 Department of Molecular Biology, Gdansk University of Physical Education, Gdansk, Poland; 8 Department of Applied Physiology, Mossakowski Medical Research Institute, Polish Academy of Sciences, Warsaw, Poland; Universidade Federal de Juiz de Fora, BRAZIL

## Abstract

**Background:**

The biological maturation (BM) analyzed by peak height velocity (PHV) and bone age (BA), and lean body mass has been associated with the strength and muscle power of young athletes. However, the ability of BM (PHV and BA) and LM markers to predict muscle strength and power in young athletes remains uncertain.

**Objective:**

The Aim was determine the predicting power of BM markers (PHV and BA) and LM in relation to muscle power of upper and lower limbs and muscle strength of upper limbs in adolescent athletes at puberty.

**Methods:**

Ninety-two adolescent athletes (both sexes; age 12.4 ± 1.02 years) were assessed for body composition by dual-energy X-ray absorptiometry (DXA). Power of upper limbs (ULP), force handgrip (HG), vertical jump (VJ) and countermovement jump (CMJ) were recorded. BM was predicted by mathematical models to estimate PHV and BA. Multilayer artificial neural network analyses (MLP’s) were used to determine the power of prediction of LM, PHV and BA on muscle power and strength of upper- and lower-limbs of the athletes.

**Results:**

LM, BA and PHV were associated with HG (r>0.74, p<0.05) and ULS (r>0.60, p<0.05) in both sexes. In both sexes BA was associated with VJ (r>0.55, p<0.05) and CMJ (r>0.53, p<0.05). LM indicated associations (r>0.60, p<0.05) with BA and with PHV (r<0.83, p<0.05) in both sexes. MLP’s analysis revealed that the LM provides > 72% of probability to predict the muscle power of upper- and lower-limbs, and the strength of the upper limbs; whereas PHV provides > 43% and bone age >64% in both female and male adolescent athletes.

**Conclusion:**

We identified that, like PHV and BA, LM is a strong predictor of low cost of both upper limbs muscle strength and upper and lower limbs power in adolescent athletes.

## Introduction

Predicting the Biological maturation (BM) in sports performance confers an essential advantage for the development and specialization of technical skills [1]. As a result of a dramatic increase in the growth factors production during puberty, morphological changes in the bone and muscle tissue show to enhance the contractile performance of skeletal muscles in speed and strength [2]. The BM is also influenced by these hormonal changes [3], although they may be modulated by environmental factors [4]. For instance, scientific reports have consistently demonstrated that individuals of a same chronological age might remarkably differ in the BM [5, 6].

To determinate the individual degree of maturation at a certain chronological age using field resources is a current challenge, regarding that the BM encloses the development of a vary of organs and systems further than bone mineralization [1–7]. In sports field, the age is a common criteria used as for gathering young athletes in categories, identifying early talents, or establishing guidelines for training prescriptions [8]. However, matching youth athletes on the basis of BM should be a more effective strategy for inclusion into competition and training strategies due to a proper exposition to the sports’ demands on specific capabilities such as muscle strength and power [7–9].

Low-cost and reliable evaluation protocols report that the BM is a relevant contributor factor for the decisions about selecting young talents in sports [3–15]. Among those specific for BM evaluation, the indirect analysis of sexual characteristics is an assessable tool, however, the method requires exposure of the young to their identification through images of genitals which might confer limitations [10–15]. Although practical, the reliability of this method might be compromised by a super estimation or underestimation when individuals are found in circumstances of embarrassment [15].

Thus, the estimation of BM by anthropometrics parameters puberty markers–as determined by the bone age (BA) by peak height velocity (PHV)–appear to be a more precise model for determination of these capabilities [11, 12]. Nevertheless, although there are protocols for BM evaluation based on BA, little is known about the relation between the BM and the main capabilities pursued in athletic performances, i.e. muscle power and strength [5–15].

We have recently reported that the PHV can estimate the lower-limbs power in young elite athletes, and is correlated with the isometric strength peak (in the handgrip test) [16]. Moreover, when BM is estimated by the BA in adolescent athletes, it correlates with both upper- and lower-limbs strength [5–17]. Furthermore, a prior study of Azimi et al. [18] showed that the Lean mass (LM) is also associated with muscle power in young weightlifters. Consonant, Raymond-Pope at al. [19] have demonstrated that the lower limbs LM is associated with lower-limbs strength in female athletes. In line, we have observed associations between BM and LM in adolescent athletes of both sex [16].

To identify clinically measurable parameters for the early forecast of young athletes in sports, in our primary study we used artificial neural networks of the multilayer perceptron type and observed that the PHV and BA are strongly associated with the muscle power (PHV: 45% - 72%, BA: 55% - 64%) [5–13]. Further, it has previously been demonstrated that young athletes with higher levels of muscle power and strength have higher concentrations of LM and a superior BM degree as compared to those with lower levels of muscle power and strength [5]. In line with this, the relation between BM and LM with isometric strength peak, as well as the muscle power in superior limbs of young athletes has already been established [13]. Nevertheless, the possibility of using LM and other BM markers, such as PHV and BA, as potential predictors of muscle power and strength in young athletes is yet to be established [5–13, 15–18].

In this study, we have tested whether the LM, as well as the PHV and the BA are reliable predictors of muscle power (of upper- and lower-limbs) and strength (of upper limbs) in adolescent athletes in puberty. The hypothesis is that LM, PHV and BA are reliable predictors of muscle strength and power in adolescent athletes.

## Methods

### Subjects

Ninety two adolescents athletes (12.4 ± 1.02 years old; 71.8% male and 28.2% female), level “V” on a scale from I to VI that classifies athletes who take part in state teams and national competitions [20], were recruited from different sportive clubs (rowing, swimming, football, Brazilian jiu-jitsu, volleyball and tennis) of Natal city, Rio Grande do Norte state–Brazil, according to the following criteria: (i) aged between 11 and 13 years old; (ii) practicing only one sport modality at least one year prior to the study; (iii) they should be registered in a their sport’s federation for at least one year. (iv) not using any type of substance that could exert an ergogenic effect (i.e. food supplements, drugs); (v) not presenting osteomyoarticular lesions in the last six months. Participants who showed any type of limitation to perform the proposed physical tests were excluded from the study.

### Ethical

Participants and their respective parents were informed about the objectives of the research and the methodological procedures being used in the study and signed informed consent and assent was obtained. This research study was approved by the Ethics and Research Committee of the Federal University of Rio Grande do Norte, Brazil (Opinion: 3.726.772), respecting the national and international ethical principles contained in the declaration of Helsinki.

### Procedures

**Fig 1:**
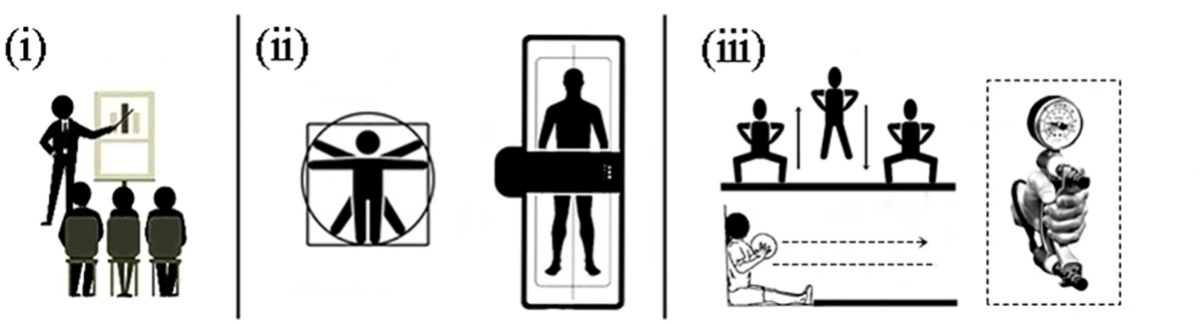
Study design—illustration of the moments and procedures used in the research. (i) Study’s explanation and Ethical consent. (ii) Day 1—Evaluation of body composition. (iii) Day 2 –Neuromuscular evaluation: muscle power (upper and lower limbs); and the peak of isometric strength (upper limbs).

This study meets the requirements of the STROBE checklist for observational studies [21]. Data collection occurred in three stages (See Fig 1).

### Anthropometry

Anthropometric assessments were performed according to the International Society of the Advancement of Kinanthropometry protocols [22]. Body mass was measured using a digital scale with an accuracy of 0.1 kg (FILIZOLA®, São Paulo, Brazil). Height and sitting height (trunk length) were assessed using a stadiometer with an accuracy of 0.1 cm (SANNY®, São Paulo, Brazil). Leg length was calculated by subtracting trunk length from height [12]. The perimeter (biceps) was measured using an anthropometric tape (SANNY®, São Paulo, Brazil), the bone diameters (Humerus and femoral) were measured using a caliper (SANNY®, São Paulo, Brazil), and skinfold (triceps) was measured by a scientific skinfold caliper (SANNY®, São Paulo, Brazil).

### Body composition

Body composition was assessed with dual energy x-ray bone densitometry (DXA) (LUNAR® / GE PRODIGY—LNR 41,990, Software enCORE version 18; United States) using specific algorithms for the pediatric population [23]. This procedure is considered one of the most reliable standard for measuring body composition [24]. Evaluations lasted 10 minutes average. DXA analyzes generated data on total lean mass, total fat mass, bone mineral density and bone mineral content were collected.

### Biological maturation assessment

The BM was estimated in relation to both the BA and the PHV [4]. For BA, used the mathematical model proposed by Cabral et al., [11]:

**Table:** 

Bone age = -11.620 + 7.004 × (Height _(m)_) + 1.226 × (Dsex) + 0.749 × (Age _(years)_) - 0.068 × (Triceps Skinfold _(mm)_) + 0.214 × (Corrected Arm Circumference _(cm)_) - 0.588 × (Humerus Diameter _(cm)_) + 0.388 × (Femoral Diameter _(cm)_)

Dsex: For male sex = 0; for female sex = 1. (m): meters. (mm): millimeters. (cm) centimeters.

It is noteworthy that to find the correct value of the corrected arm perimeter in the equation of Cabral et al., [11], just use the following equation:

$$\begin{array}{c} Corrected\;Arm\;Circumference_{\left(cm\right)}=\\ Contracted\;biceps\;circumference_{\left(cm\right)}‐(Triceps\;Skinfold_{\left(mm\right)}/10) \end{array}$$

(mm): millimeters. (cm) centimeters.

The BM was then estimated by the equation [15]: Bone age—Chronological age. Results were used to qualify the participants in three stages of maturation: Delayed (Results ≤-1), Synchronized (Results between -1 and 1) and Accelerated (Results ≥ 1).

The PHV was estimated by the mathematical models proposed by Mirwald et al., [12]:

**Table:** 

Maturity offset in males = −9.236 + [0.0002708 × (Leg length _(cm)_ × Trunk Height_(cm)_)] + [−0.001663 × (Age_(years)_ × Leg length_(cm)_)] + [0.007216 × (Age _(years)_ × Trunk Height_(cm)_)] + [0.02292 × (Weight_(kg)_ / Height_(cm)_) × 100]
Maturity offset in females = −9.376 + [0.0001882 × (Leg length_(cm)_× Trunk Height_(cm)_)] + [0.0022 × (Age_(years)_× Leg length_(cm)_)] + [0.005841 × (Age _(years)_ × Trunk Height _(cm)_)] − [0.002658 × (Age_(years)_× Weight_(kg)_)] + [0.07693 × (Weight_(kg)_ / Height_(cm)_) × 100]

(cm) centimeters. (kg): kilograms.

Results were used to classify the participants in three maturity statuses [12]: 1) Pre-PHV = maturity offset <-1; 2) circum-PHV = maturity offset >-1 and <+1; and 3) Post- PHV = maturity offset >+1.

### Upper limbs strength and power

Using a hydraulic dynamometer (JAMAR^®^, Cambuci, Brazil), participants were seated on a bench adjusted to their height and holding the dynamometer with the elbow flexed at a 90° angle. Three maximum grip contractions (3-second with recovery periods of 60 s) with both hands were performed. The best performance was considered for statistical analysis [25]. Then after, seating on the floor with the back against a wall at 90° and knees extended, the participants threw a 2-kg medicine ball (Ax Sports^®^, Tangará, Brazil) as far as possible, using both hands from the level of their sternum—trunk movement was not allowed. Three attempts with 3-min recovery were performed and the best result was considered for statistical analysis [26].

### Lower limbs power

The performance of the lower limbs was attained by tests of vertical jump (VJ) and countermovement jump (CMJ) using a force platform (CEFISE^®^, São Paulo, Brazil) and protocols established by Forza & Edmundson [27]. Before the evaluations, the subjects performed a jump of each type to familiarize themselves with the tests, seeking to reduce errors during the execution of the protocols. Then, starting from an orthostatic position, held for three seconds, with the knees flexed at approximately 90° and the hands fixed on the waist, the subjects were instructed to perform a maximal vertical jump. For CMJ analysis the same recommendations were adopted, however, the volunteers performed a squat followed by the jump. A 10-minute recovery interval was observed between VJ and CMJ. For both tests, three attempts were made, interspersed with 60s of passive recovery and the best attempt was used for data analysis.

### Statistics procedures

#### Analysis for sample size

The calculation of the total sample for this research was carried out a priori, based on previous studies [6–16] the variables considered in the sample calculation were: Lean Mass, PHV, BA, ULS, HG, VJ and CMJ. A significance of α <0.05 and a β = 0.80 was taken into account, for all the variables mentioned above the power stipulated a priori (Using "T" statistics) for the minimum sample of 25 subjects per group was >0.80. We emphasize that the analysis were repeated a posteriori (details in the results section). For the analysis of sample power, open source software was used G* Power® (Version 3.0; Berlin, Germany).

#### Data normality test and comparisons between groups

Normality was tested using the Kolmogorov-Smirnov tests and z-score for asymmetry and kurtosis (-1.96 to 1.96). Comparisons between groups [male X female] were made using Student’s independent t test.

#### Association analysis and analysis of technical error of measures

The associations of the data were made by the Pearson test. The magnitude used was the one proposed by Schober, Boer and Schwarte [28]: Insignificant: r <0.10; Weak: r = 0.10–0.39; Moderate: r = 0.40–0.69; Strong: r = 0.70–0.89; Very strong: r = 0.90–1.00. For the technical error of anthropometric measurements, the following magnitude was used: acceptable for skinfolds ≤ 5.0%; acceptable for other anthropometric measurements ≤ 1.0% [29].

#### Analysis of the power of prediction between variables

Non-linear artificial neural networks of the multilayer perceptron type (MLP’s), were programmed in “R” language, with the objective of estimating the power of the prediction of the independent variables (LM, PHV and BA) in relation to the associations with the dependent variables (HG, ULS, VJ and CMJ). MLP’s were programmed with backpropagation algorithms to adjust synaptic weights [30]. The sample was divided based on sex (male and female) and in both groups, 70% of the sample was used for training and 30% for MLP’s tests in 10,000 execution times [30]. For cross-validation, the sample was alternated between MLP’s training and testing until all data had passed through both conditions, this procedure was repeated for ten consecutive times and the average results of the ten repetitions in relation to the power of the importance of the independent variables in relation to associations with dependent variables, were taken as the final results [30].

All analyzes were performed using open source software R (version 4.0.1; Foundation for Statistical Computing®, Vienna, Austria) considering the significance of p <0.05.

## Results

Table 1 shows the participants characteristics (there was no loss of data). The margin of error pointed out for the sample size was 4.87%, being <5% (Suggesting >95% reliability for the analyzes performed). Females were more advanced in BA stage and PHV and had greater fat mass than males (p<0.05). In contrast males had greater LM and bone mineral density (p<0.05).

**Table 1:** Sample characterization in relation to the characteristics of puberty, body morphology and neuromuscular tests.

Variables	Male	Female
Number	66	26
Age (years)	12.3 ± 1.03	12.6 ± 1.02
Bone age (years)	12.8 ± 2.03	12.6 ± 2.16
Bone age Stage	0.55 ± 1.40	*2.11 ± 1.69
Peak Height Velocity	0.72 ± 1.96	*1.31 ± 1.68
Height (cm)	160.8 ± 13.1	158.6 ± 9.70
Body weight (kg)	52.6 ± 14.9	50.8 ± 11,9
Fat mass (kg)	11.8 ± 5.97	*15.1 ± 6.07
Lean mass (kg)	*39.2 ± 11.0	33.7 ± 6.64
Bone mineral content (g)	*2.30 ± 0.84	1.79 ± 0.78
Bone mineral density (g/cm^2^)	1.48 ± 0.46	1.55 ± 0.47
Handgrip (kgf)	26.6 ± 10.0	26.6 ± 10.1
Upper limbs power (cm)	3.38 ± 1.23	3.46 ± 1.22
Vertical jump (cm)	31.9 ± 8.97	31.4 ± 9.56
Countermovement jump (cm)	34.1 ± 9.93	33.3 ± 10.5

*p = 0.01. cm = centimeters. kg = kilograms. g = grams. g/cm^2^ = grams per square centimeter. kgf = kilograms force per square centimeter.

In addition, the posterior sample calculations indicated sampling power> 0.80 for both sexes (considering the variables LM [male: 0.99, female: 0.99], PHV [male: 0.80, female: 0.80], BA [male: 0.94, female: 0.82], ULS [male: 0.99, female: 0.95], HG [male: 0.99, female: 0.99], VJ [male: 0.95, female: 0.90] and CMJ [male: 0.95, female: 0.92]).

Table 2 shows associations between LM and muscle performance. Whilst males showed significant positive mild to moderate relationships with measures of strength, in females these relationships were only observed in ULS and handgrip performances. Bone age in both sexes was associated with neuromuscular variables and lean mass. PHV was associated with neuromuscular variables in the upper limbs and lean mass in both sexes. Regarding the lower limb neuromuscular variables, PHV was significantly associated only in males.

**Table 2:** Matrix of associations of the neuromuscular tests with lean mass, bone age and peak height velocity and of lean mass with bone age and peak height velocity.

Tests	Male		Female	
	r	CI 95%	r	CI 95%
	Associations with lean mass			
Handgrip	0.85***	[0.80; 0.90]	0.83***	[0.78; 0.85]
Upper Limb Power	0.73***	[0.69; 0.77]	0.75***	[0.71; 0.80]
Vertical Jump	0.57**	[0.50; 0.59]	0.11	[0.03; 0.15]
Countermovement Jump	0.59***	[0.58; 0.65]	0.16	[0.10; 0.20]
	Associations with bone age			
Handgrip	0.80**	[0.77; 0.86]	0.63**	[0.60; 0.71]
Upper Limb Power	0.74**	[0.73; 0.80]	0.69***	[0.67; 0.73]
Vertical Jump	0.67**	[0.60; 0.70]	0.55*	[0.45; 0.56]
Countermovement Jump	0.71***	[0.71; 0.80]	0.53*	[0.49; 0.55]
Lean Mass	0.81***	[0.77; 0.89]	0.60*	[0.55; 0.62]
	Associations with Peak Height Velocity			
Handgrip	0.87***	[0.80; 0.91]	0.74***	[0.71; 0.80]
Upper Limb Power	0.74**	[0.70; 0.78]	0.79***	[0.76; 0.84]
Vertical Jump	0.56**	[0.52; 0.60]	0.32	[0.28; 0.40]
Countermovement Jump	0.54***	[0.51; 0.58]	0.35	[0.25; 0.37]
Lean Mass	0.84***	[0.82; 0.89]	0.83***	[0.79; 0.87]

*** p<0.0001.

** p = 0.0001.

*p = 0.01. CI 95% = confidence interval of 95%.

**Fig 2:**
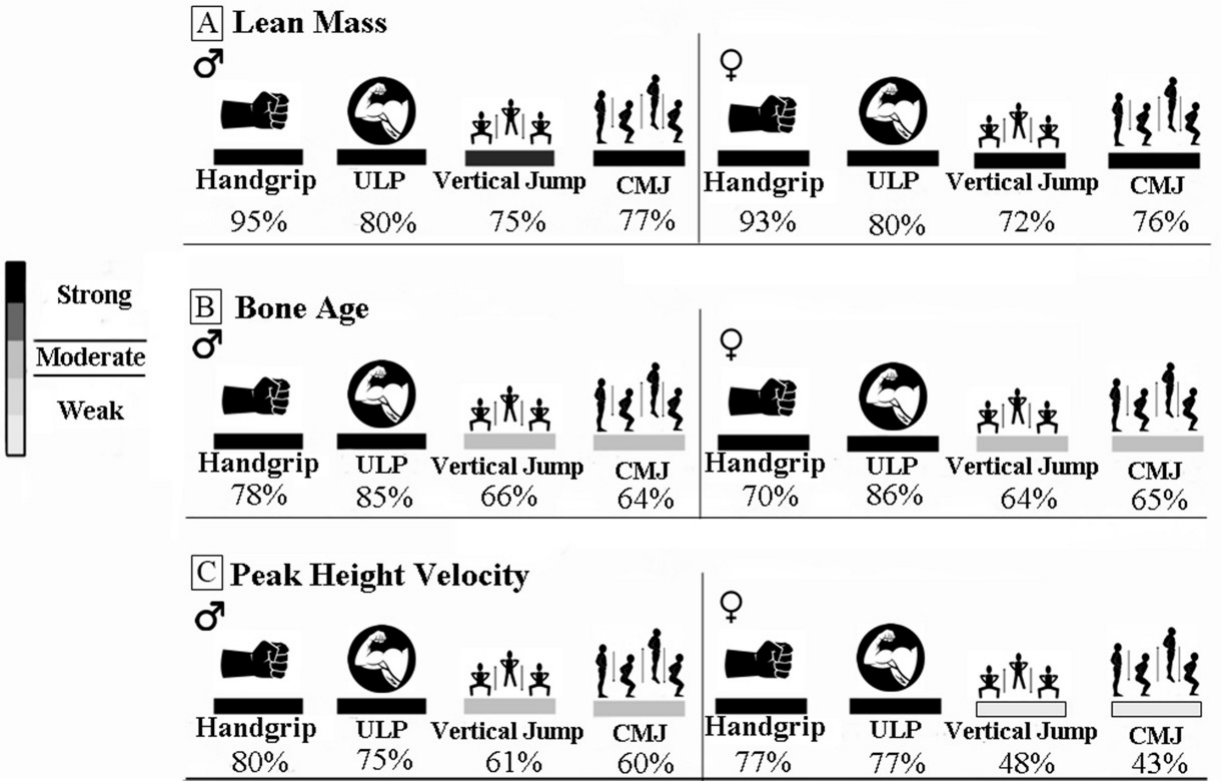
Analysis of MLP’s to indicate the capacity of lean mass, bone age and peak height velocity in predicting the neuromuscular variables of the present study. % = Prediction percent. ♂ = Male sex. ♀ = Female sex. ULP = Upper limbs power. CMJ = Countermovement jump. A = Lean mass as predictor of handgrip, upper limbs power, vertical jump and countermovement jump. B = Bone age as predictor of handgrip, upper limbs power, vertical jump and countermovement jump. C = Peak height velocity as predictor of handgrip, upper limbs power, vertical jump and countermovement jump.

Analyzes of MLP’s indicated that in both sexes the lean mass indicated the ability to predict neuromuscular variables in probabilities above 70%. Bone age indicated prediction probabilities above 64% for neuromuscular variables and PHV pointed probabilities above 43% for the prediction of neuromuscular variables in the sample (Fig 2).

## Discussion

The aim of this study was to determinate the power of LM, PHV and BA to predict the power of upper and lower limbs and strength upper limbs in adolescent athletes. We highlight, that the sample of young’s athlete is a rare audience, being composed mostly of young males, which causes disparities between males and females in the samples of young athletes in different sports [20]. This fact corroborates with the present study, where the male sample was large than the female sample. On the other hand, in present study cluster segregation analyses were performed considering both genders (male and female), i.e., the power and quality of the results were not affected by the difference in sample size. Moreover, it is possible to state that the sample size for both sexes was within the parameters established a priori by the sample calculation (See section "Analysis for Sample Size" in topic "Statistics Procedures").

The hypothesis of the present study that len mass (LM), peak height velocity (PHV) and bone age (BA) are reliable predictors of muscle strength and power in adolescent athletes was confirmed through the results: (i) LM and PHV associate with upper limbs strength and power in adolescent athletes. Only in males, the LM and PHV were not associated to the lower-limbs power. (ii) BA associate with both strength in upper limbs and power in upper and lower-limbs of adolescent athletes. (iii) PHV and BA are associated with LM in all sample. (iv) Further MLP’s analysis revealed that the LM provides > 72% of probability to predict the muscle power of upper- and lower-limbs, and the strength of the upper limbs; whereas PHV provides > 43% and bone age >64% in both female and male adolescent athletes.

In consonance with Pourmotahari [31] thesis of the influence of BM on components of muscle performance, it was observed that a secondary factors as the LM interferes in the relationship between neuromuscular performance and BA. In a previous study, Gouvea et al. [14] have observed that BM correlates with strength in upper limbs of young soccer players aging from 14 to 17, and that the levels of LM might have had an interaction with this strength. Later on, Dantas et al. [32] study has described that BM has a significant relationship with the strength of upper and lower limbs in adolescent rowers of both sexes, aging from 8 to 14 years. The present study shows that, in addition to PHV and BA, LM is an excellent predictor of upper limb muscle strength and power, and lower limb muscle power, in adolescent athletes.

This finding corroborates those of Alzimi et al. [18] study, in which the authors have identified that the total body mass as well as the LM relate to muscle power in young athletes aged 16 years. Pinto et al. [6] in your study showed that adolescents aging from 10 to 13 years with higher total body mass showed better performance of upper limb muscle power and lower limb when compared to those with lower total body weight.

The analysis of MLP’s enabled the current study to identify that LM is a strong predictor of the muscle power the upper and lower limbs in adolescent athletes, and also of the strength in the upper-limbs. Furthermore, we have previously demonstrated in studies that while BA and PHV are strong predictors of the upper-limbs strength, secondary factors such as steroid hormones and LM seem to be more effective to predict the performance of lower-limbs [5–33]. Associations between LM and strength in young adults (20.4 ± 1.4 years) have been previously reported by Raymond-Pope et al. [19].

This study reports that both BM and LM parameters are strong predictors of the strength of the upper-limbs, and muscle power. MLP’s analysis revealed that either the LM or the parameters used for determination of BM (i.e., PHV and BA) show a power greater than 70% for prediction of strength and power performance of upper limbs. LM and BA showed a prediction power greater than 64% for the power of lower-limbs, and PHV showed > 60% power for the male subjects and > 50% for the female’s. Interestingly, these results diverge from those in our previous study in which PHV presented a prediction power greater than 70% for the strength of upper and lower limbs in female subjects [13]. Such difference, might express a sport- specificity bias when observing that the first study was addressed to female non-athletes, whereas in the current study, the participants were well familiarized with the technique for the execution of the tests.

Although the study has relevance in the results, some limitations were listed: (i) The research design (observational), which does not allow a cause and effect relationship to be established. (ii) Biological maturation was obtained based on predictive models, where the results based on clinical tests may differ (i.e., hand and wrist x-rays and longitudinal monitoring of the onset of puberty in those evaluated).

## Conclusion

Based on the results of this research, it was possible to conclude that markers of biological maturation and lean mass index are strong predictors of upper limb muscle strength and upper and lower limb power in adolescent athletes of both sexes. Additionally, the mentioned parameters can be reliable and low cost tools to help in the process of orientation and identification of the potential of young athletes.

## Supporting information

S1 Graphical abstract (TIF)
