# Supplementary figures and images for: Lean mass and biological maturation as predictors of muscle power and strength performance in young athletes
Source: PLoS One. 2021 Jul 12;16(7):e0254552. doi: 10.1371/journal.pone.0254552 (PMC8274902; doi:10.1371/journal.pone.0254552)

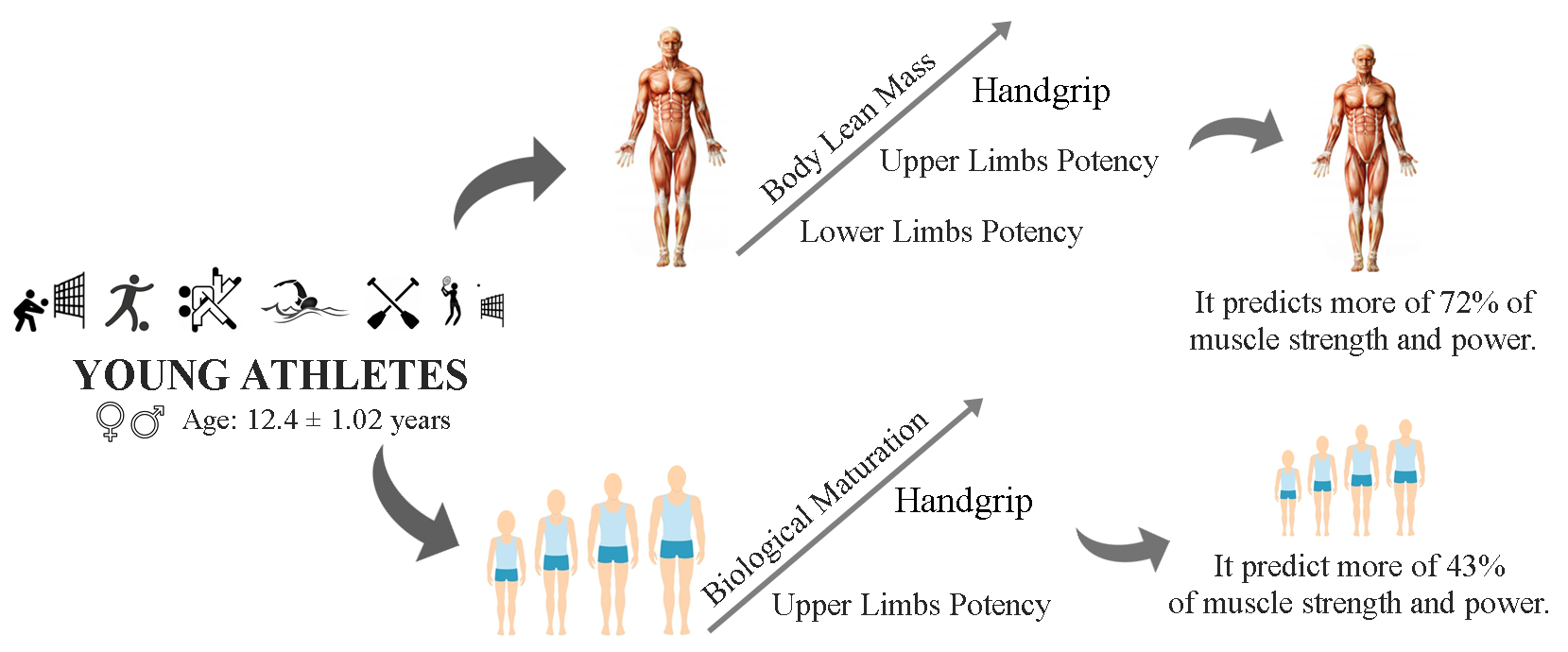

Supplement: S1 Graphical abstract — (TIF) [file pone.0254552.s001.tif]
